# National priorities in the power system transition to net-zero: No one size fits all

**DOI:** 10.1016/j.isci.2022.105260

**Published:** 2022-10-02

**Authors:** Yoga Wienda Pratama, Piera Patrizio, Niall Mac Dowell

**Affiliations:** 1 Centre for Environmental Policy, Imperial College London, London SW7 1NE, UK; 2 The Sargent Centre for Process Systems Engineering, Imperial College London, London SW7 2AZ, UK

**Keywords:** Energy resources, Energy policy, Energy systems, Energy management, Energy modeling

## Abstract

Net-zero emissions targets are increasingly being adopted globally. However, there is a disconnect between policy mechanisms, that primarily focus on incentives to reduce emissions, and technological requirements to achieve this aim. In this context, an absence of CO_2_ removal incentives effectively precludes complete decarbonization, while potentially increasing the cost. Here, we quantify the effectiveness of carbon tax, negative emissions credit, and technology improvements in delivering net-zero targets cost-effectively in the UK, Poland, Texas, and Wyoming power systems. We show that the combination of a carbon tax and negative emissions credit is critical to reaching net-zero targets. From the techno-economic perspective, while all cost reduction is welcome, a further cost reduction of renewable energy is uniquely valuable in minimizing the transition cost. However, even with the availability of electricity storage, dispatchable and low-carbon thermal plants are key to cost-effectively maintaining system reliability, regardless of the costs of other alternatives.

## Highlights

• System’s characteristics are keys to net-zero (NZ) transitions strategy
• NZ transitions in power systems require carbon tax and negative emissions credit
• The value of arbitrary technology cost or efficiency targets is system-specific
• System’s characteristics, policy mechanisms, and technical targets are interrelated

## Graphical abstract

**Figure:**
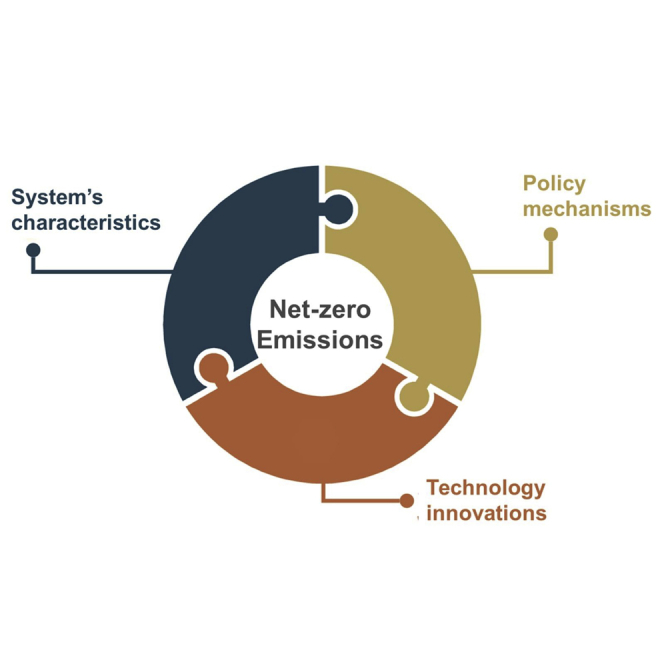

## Teaser

Energy resources; Energy policy; Energy systems; Energy management; Energy Modelling;

## Introduction

The global energy system in the 20^th^ century relied near-exclusively on fossil fuels. Over this period, fossil-based power generation HHV-based efficiency substantially increased up to 5-fold, from 10% in the original coal-fired in the early 1900s to 46% in IGCC power plants, while natural gas plant’s efficiency increased from 20% when first introduced in the 1940s to over 60% in a modern gas-fired power station (Smil, 2000; Kaneko, 2016). Similarly, within the same time frame, the capital cost of coal-fired power plants dropped from £ 5,000/kW to £ 1,600/kW (McNerney et al., 2011) and the capital cost of CCGT decreased from £ 1,100/kW to £ 550/kW between the 1980s and the present days (McDonald and Schrattenholzer, 2002; BEIS, 2016). As the system was homogenous, such improvements are greatly advantageous. As an illustration, the range of electricity prices in the US dropped from 118–740 £/MWh to 48–106 £/MWh between the 1920s and 1970s (USCB, 1976). From that point, however, such cost figures remained mainly constant, despite the continued improvements (US. & EIA, 2021).

The 1970s oil shocks prompted the development of alternative technologies, with the aim of protecting the economy from the perceived volatility of fossil energy prices (Sovacool, 2013; Mitchell, 2016; Pratama and Mac Dowell, 2020). From the early 1990s, the development of non-fossil energy technologies accelerated further as climate change emerged as a prominent issue in energy system development (UNFCCC, 1992; UNFCCC, 1998; UNFCCC, 2015). Between 1970 and 2018, global nuclear capacity increased from 18 to 397 GW (International Atomic Energy Agency (IAEA), 2019), while renewable energy sources (RES) reached 83% of the net annual addition of global power-generating capacity in 2020 (IEA/OECD, 2018; REN21, 2021), thus increasing the diversity of energy supply. As a result, energy systems became more resilient to potential shocks. This diversity, however, also intrinsically weakens the value of future individual technology innovations, while synergistic technology improvements unique to each system become increasingly important.

At the time of writing, an increasing number of countries have committed to achieving carbon neutrality targets in their energy systems by 2050–2060, including the UK (UK Parliament, 2008; UK Parliament, 2019), the US (White House, 2021), the EU (European Commission, 2020), and South Korea (The Government of the Republic of Korea (2020), 2050), among 134 countries worldwide (ECIU, 2021). However, within this context, the prevailing policy framework is still characterized by monothetic approaches based on supportive policy mechanisms (carrots), such as renewable energy feed-in-tariffs or subsidies, or on punitive measures (sticks), such as carbon taxes or cap-and-trade mechanisms. In particular, carbon pricing is often regarded as the most effective measure to identify and incentivize the least cost way to net-zero (World Bank, 2019; Dennig et al., 2015; Stiglitz et al., 2017). However, while the existing portfolio of options is sufficient to sustain the rapid deployment of renewable energy and reduce CO_2_ emissions from thermal power plants, its effectiveness significantly reduces as emissions are reduced, and it does not incentivize the essential removal services that negative emissions technologies (NETs) can provide (Daggash and Mac Dowell, 2019; Patt and Lilliestam, 2018; Rosenbloom et al., 2020; Bednar et al., 2019).

**Figure 1:**
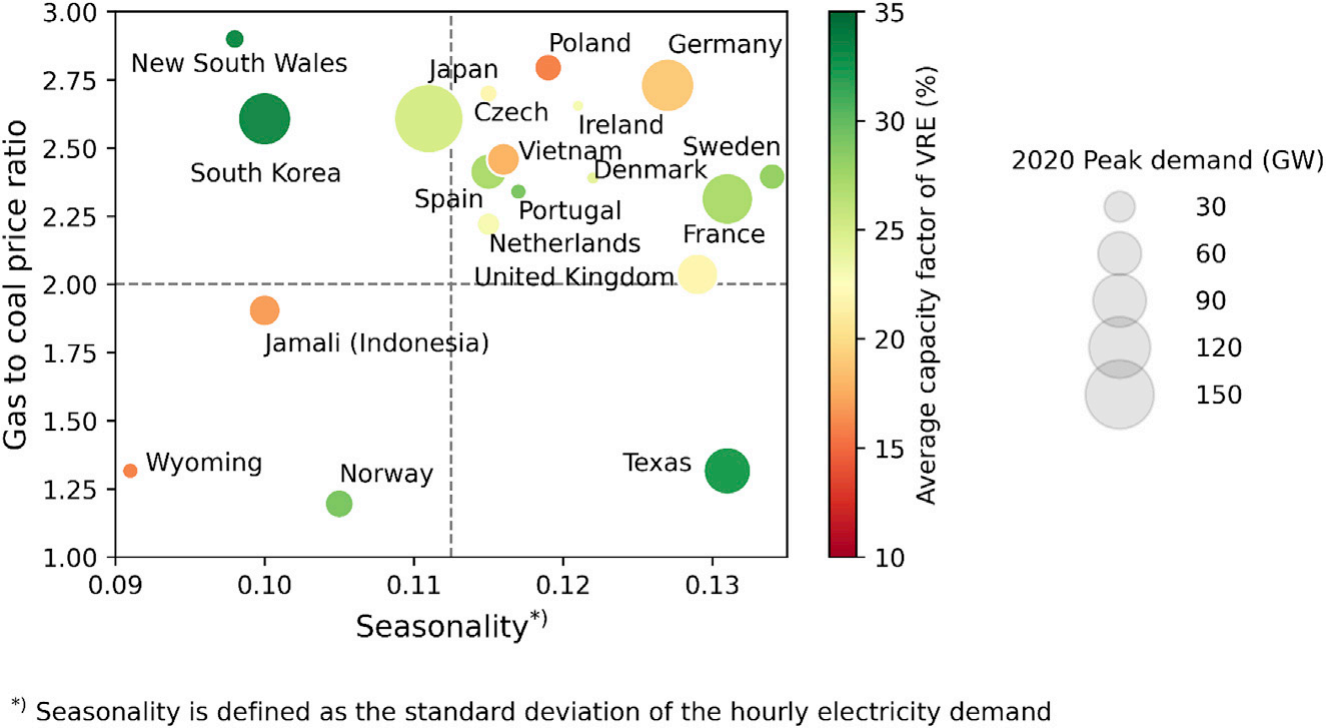
Key characteristics of different power systems

From the system perspective, the rapid penetration of renewable energy highlights the value of flexibility and ancillary services provided by firm capacity (Pratama and Mac Dowell, 2020; Lamont, 2008; Bouzguenda and Rahman, 1993; US. & EIA,; IEA, 2019), which could be cost-effectively provided by low-carbon thermal plants (Heuberger et al., 2017; Lehtveer and Fridahl, 2020; Zappa et al., 2019). However, by focusing on technologies in isolation (BEIS, 2018), existing policy paradigms prioritize cost reduction measures as the key driver for low-carbon technology deployment, with insufficient, if any, attention to the role that each technology might play in the overall system. Moreover, as illustrated in Figure 1, every distinct energy system is characterized by a unique set of characteristics, including seasonality of power demand, availability of renewable energy, fuel prices, and system size. The unique combination of those characteristics imposes different challenges to the decarbonization of a given system and can significantly affect the role and value of each technology therein (Pratama and Mac Dowell, 2019). Moreover, the future electricity system will be strongly influenced by various factors that remain uncertain, namely: technology capital costs, fuel prices, and technical performances such as thermal plant and storage round-trip efficiencies. Hence, although several studies have identified technology improvement priorities (Heuberger et al., 2017; Sepulveda et al., 2021; Frew et al., 2021) under a specific pathway, there is a lack of attempts to robustly identify these priorities against future uncertainty. Most importantly, there have been negligible attempts to explore the disconnect between policy mechanisms and technological improvement priorities which may have a long-term impact on net-zero transitions, including for the electricity system.

This contribution attempts to close this gap by presenting a new lens through which the net-zero transition problem can be addressed. Rather than considering supportive and punitive policy measures in isolation, we adopt a carrot and stick approach to identify likely portfolios of low carbon technologies that are key to net-zero (what), while accounting for the specific role these technologies will play (how) in a variety of different energy systems (where). Furthermore, we also identify technology improvement priorities that are likely to reduce the range of transitions’ costs further. In this study, we use the UK, Poland, ERCOT Texas (USA), and PacifiCorp East (PACE in Wyoming/USA) as power system archetypes with distinct characteristics and existing targets.

In this study, we identify systems with different key characteristics; this includes 1) fuel prices in the y axis are shown in terms of gas to coal price ratio, 2) seasonality of the electricity demand shown in the x axis which is quantified using the standard deviation of the hourly electricity demand, 3) availability of renewable energy is shown by the color of the bubble which is quantified using the average capacity factor of solar photovoltaic, onshore and offshore wind, and 4) the system’s size in term of peak demand represented by the size of the circles, respectively.

## Results and discussion

### Policy dimension: Sticks and carrots

**Figure 2:**
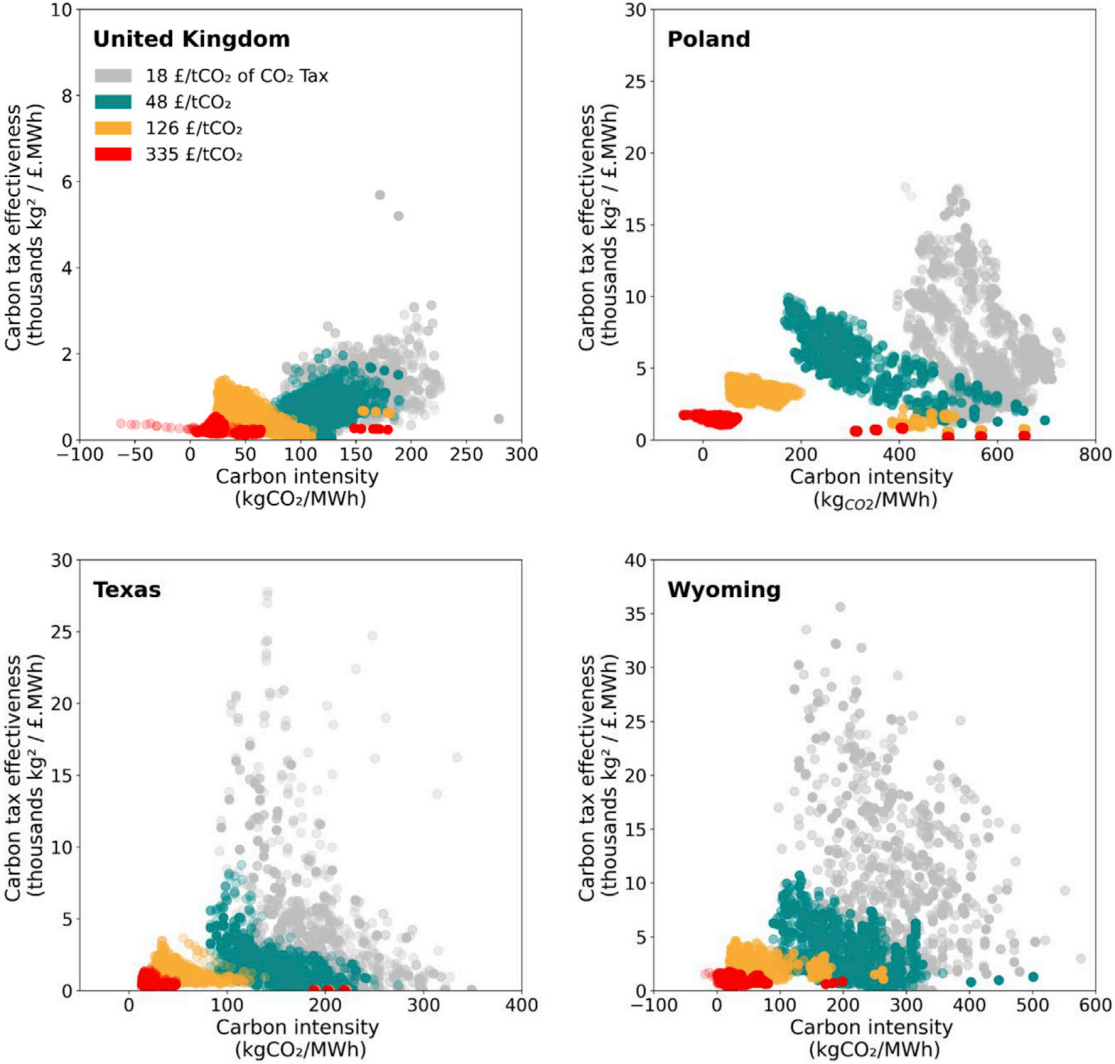
Effectiveness of carbon tax in different power systems archetypes This figure shows the range of carbon intensities (x axis) vs. carbon tax effectiveness (y axis) of (a) the United Kingdom, (b) Poland, (c) Texas, and (d) Wyoming. The plots’ colors show different ranges of results for 18, 48, 126, and 335 £/tCO_2_ carbon tax, respectively. The effectiveness of carbon tax reduces significantly as the system’s carbon intensity declines. From 100 to 740 kgCO_2_/MWh, the effectiveness of carbon taxes in the range of £18–48/tCO_2_ is very high (5–15 thousand kg^2^/£.MWh), especially in the cases of Poland, Texas, and Wyoming. However, to advance the decarbonization lower than 100 kgCO_2_/MWh requires a higher carbon tax as the effectiveness drops to 0.3–2 thousand kg^2^/£.MWh, implying a new mechanism may be required.

Carbon pricing is a widely recognized mechanism to impose a penalty on CO_2_ emissions (World Bank, 2019; Dennig et al., 2015; Stiglitz et al., 2017). It is particularly effective in carbon-intensive systems, characterized by large point-source emissions. Here, we define carbon tax effectiveness as carbon intensity reduction that can be achieved in the system per unit of the carbon tax imposed. As can be observed from Figure 2, in systems with carbon intensity (CI) higher than 100 kgCO_2_/MWh, the effectiveness of £18/tCO_2_ and £48/tCO_2_ carbon tax is around 5–15 thousand kg^2^/£.MWh, *i*.*e*., every £1/tCO_2_ of carbon tax imposed in the system leads to reductions in the system carbon intensity in the range of 5–15 kgCO_2_/MWh. However, the instrument rapidly becomes ineffective once system carbon intensity approaches the 50–100 kgCO_2_/MWh range, that is a system with high levels of wind and solar PV deployment, nuclear, and CCS-equipped power plants (Daggash and Mac Dowell, 2019; Daggash et al., 2019).

**Figure 3:**
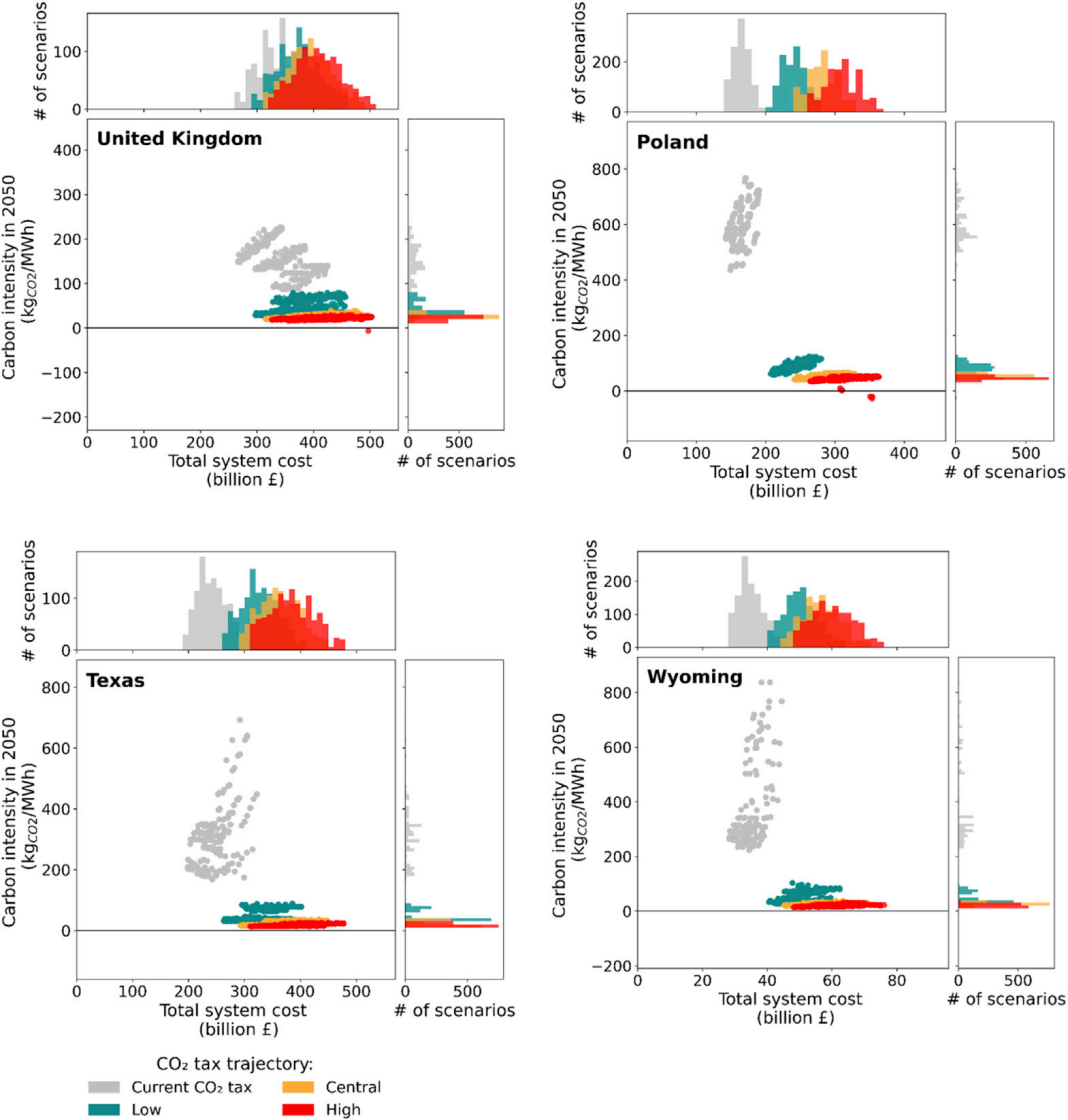
Economic and environmental impact of different carbon tax trajectories without negative emissions credit This figure presents the range of total system costs (x axis) vs. carbon intensities in 2050 (y axis) in the United Kingdom, Poland, Texas, and Wyoming. The colors of the plots, gray, green, amber, and red, show a different range of results for current, low (133 £/tCO_2_), central (236 £/tCO_2_), and high (334 £/tCO_2_ in 2050) carbon tax, respectively. See Table S6 in the supplemental information for more details. Without negative emissions credit, imposing a low carbon tax alone can significantly decrease the range of carbon intensity in 2050 from 90–820 kgCO_2_/MWh to 30–125 kgCO_2_/MWh. Increasing the carbon tax from low to central or high value, however, is not efficient as this can dramatically increase the range of total system costs with only a marginal reduction of the systems’ carbon intensities to 18–70 kgCO_2_/MWh.

**Figure 4:**
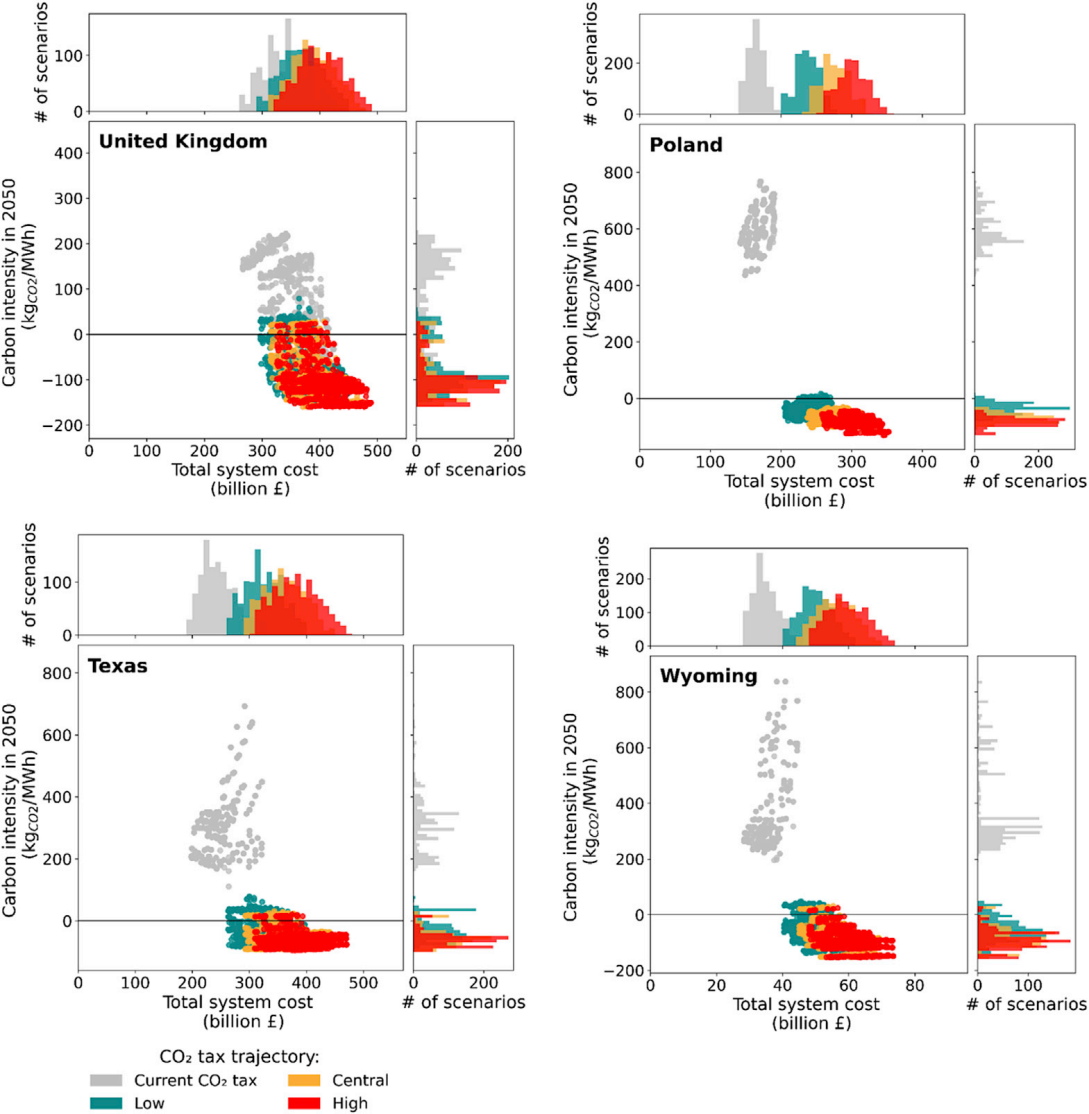
Economic-environmental impact of different carbon taxes with central negative emissions credit The range of total system costs (x axis) vs. carbon intensities in 2050 (y axis) of the United Kingdom, Poland, Texas, and Wyoming for current (gray), low (green), central (amber), and high (red) carbon tax. Combining a low or central carbon tax with central negative emissions credit is likely to deliver the net zero emissions target with greater than 80% probability. Systems with expensive natural gas such as the UK and Poland require only a low carbon tax to achieve the target by driving the deployment of CCS-equipped plants to 16%–78% of peak load compared to none in the BAU scenario. Small-size systems with less seasonal demand such as Wyoming also only require a low carbon tax to promote nuclear power plants (12%–14% of peak load) and some CCGT-CCS (21%–76% of peak load) for providing flexible capacity. Access to cheap natural gas, however, requires Texas to impose the central carbon tax to promote more RES and CCGT-CCS to provide more flexible capacity for the systems. With a low carbon tax, CCGT-CCS is deployed only 6%–14% of peak load by 2050 as opposed to 48%–72% of peak load if the central carbon tax is imposed, that is the scenario that can deliver the net-zero emissions target under the central carbon tax.

As shown in Figure 3, a carbon tax alone is insufficient to achieve complete power system decarbonization, with outcomes for imposing a low carbon tax apparently limited to a 65%–88% reduction in carbon intensity compared to a business as usual (BAU) scenario. Beyond this point, a 250% increase in the carbon tax, *i*.*e*., from £133/tCO_2_ (low) to £334/tCO_2_ (high) in 2050, achieves only marginal reductions in carbon intensity, while simultaneously leading to a 22%–61% increase in total system costs relative to BAU. However, without a carbon tax, it is exceptionally unlikely to decarbonize the system regardless of the value of the negative emissions credit provided, as shown in Figure 4.

Figure 3 also reveals that the economic impact of increasing carbon tax levels is driven by region-specific factors. Owing to a combination of abundant renewable energy, and the prohibition on new unabated coal power, a £334/tCO_2_ carbon price in the UK will result in 11%–20% higher total system costs (TSCs) compared to the BAU’s range. Conversely, TSCs increase of 46%–71% and 67%–80% is observed in Texas and Wyoming, respectively, owing to the switch from cheap coal (4.8–5.9 £/MWh) to natural gas (7.4–19.8 £/MWh). Distinct from the other systems, Poland enjoys cheap and abundant coal reserves but significantly more expensive natural gas prices, *i*.*e*., ∼270% of coal price on a thermal energy basis. Current national policies (The Ministry of Energy of Poland, 2018; The Ministry of State Assets of Poland, 2019) also ban further deployments of onshore wind. As a result, imposing high levels of the carbon tax to enforce the decarbonization of the power system in Poland increases costs by 88%–104% relative to the BAU case.

Relying exclusively on the carbon tax to achieve the net-zero targets, therefore requires a combination of favorable conditions, such as the availability of highly efficient thermal plants (*e*.*g*., ∼62% HHV and 55% HHV efficient for CCGT and CCGT with CCS, respectively), high fossil fuel prices, and low-cost wind and solar power, and low-cost biomass. These specific circumstances allow more renewables and CCS-equipped plants to be installed to lower the system’s residual emissions while carbon dioxide removal (CDR) technologies, BECCS offsets the emissions.

While being valuable to cost-effectively provide dispatchable and flexible capacity, compensating for the residual emissions from CCS technologies requires carbon dioxide removal (CDR) services, which are not incentivized by simply raising carbon taxes. Rather, achieving the required level of power systems decarbonization requires a combination of an emissions penalty and a mechanism to incentivize removals.

To achieve net-zero targets in a cost-effective manner, securing sufficient levels of CDR while minimizing the system’s residual emissions is key. As can be seen in Figure 4, a greater than 90% probability of reaching net-zero implies the introduction of a central negative emissions credit, owing to the need for deploying capital-intensive BECCS. By contrast, the level of carbon tax required to minimize the residual system emissions are case-specific depending on the value of carbon-emitting technologies. In systems with highly seasonal demand, such as the UK and Texas, a substantial amount of flexible and low-cost capacity, particularly from CCGT and OCGT, is inherently required. On the other hand, Texas and Wyoming are endowed with cheap natural gas. Hence, the punitive effect of the low carbon tax in the UK, Texas, and Wyoming is downgraded and requires the systems to implement a central or high carbon tax to put CCS investment ahead of unabated technologies. As can be observed in Figure 4, the probability of meeting the target is 85% for the UK, 78% in Texas, and 87% in Wyoming for scenarios under the low carbon tax. These probabilities are increased to 93%, 90%, and 97% for the UK, Texas, and Wyoming, respectively. By contrast, Poland’s power system is operating with a less seasonal load profile and high natural gas prices. Accordingly, technologies with low operating costs under carbon tax mechanisms to allow cost-effective operation at a higher capacity factor, such as CCGT-CCS and nuclear, are selected over unabated fossil plants. Hence, the combination of the low carbon tax and central negative emissions credit is already sufficient for Poland to decarbonize its power system at a 96% probability.

Achieving the net-zero target with a combination of the low carbon tax and central negative emissions credit in Poland implies a complete phase-out of coal from its current 68% capacity share. Hence, the capacity of natural gas-based technologies increases from 1.1 GW to 12–26 GW between 2020 and 2050. Combined with the existing ban on new onshore wind farms, this leads to the deployment of comparatively expensive offshore wind, thus increasing system costs by 45%–47% relative to the BAU.

The combination of the central carbon tax and negative emissions credit might compensate for such dramatic cost increases by tempering the negative socio-economic impacts associated with the closing of the domestic coal industry. Under such a policy paradigm, Poland retains 26% of its existing coal power capacity while simultaneously boosting the domestic biomass industry for BECCS (Patrizio et al., 2020). However, additional costs of 69%–73% of BAU’s range are required to upgrade and operate its unabated coal fleet to CCS-equipped ones.

### Technology dimension: Cost reduction vs efficiency improvement

In the discussion above, we show that the combination of the central carbon tax and negative emissions credit is sufficient to cost-effectively promote the deployment of low carbon technologies and incentivize CO_2_ removal services via BECCS deployment to decarbonize the systems. This combination dictates the role each technology will play in the system and, accordingly, determines key improvements each technology will need to reduce the cost of delivering the net-zero targets further. Hence, this section identifies key technology improvements that should be prioritized within each power system.

**Figure 5:**
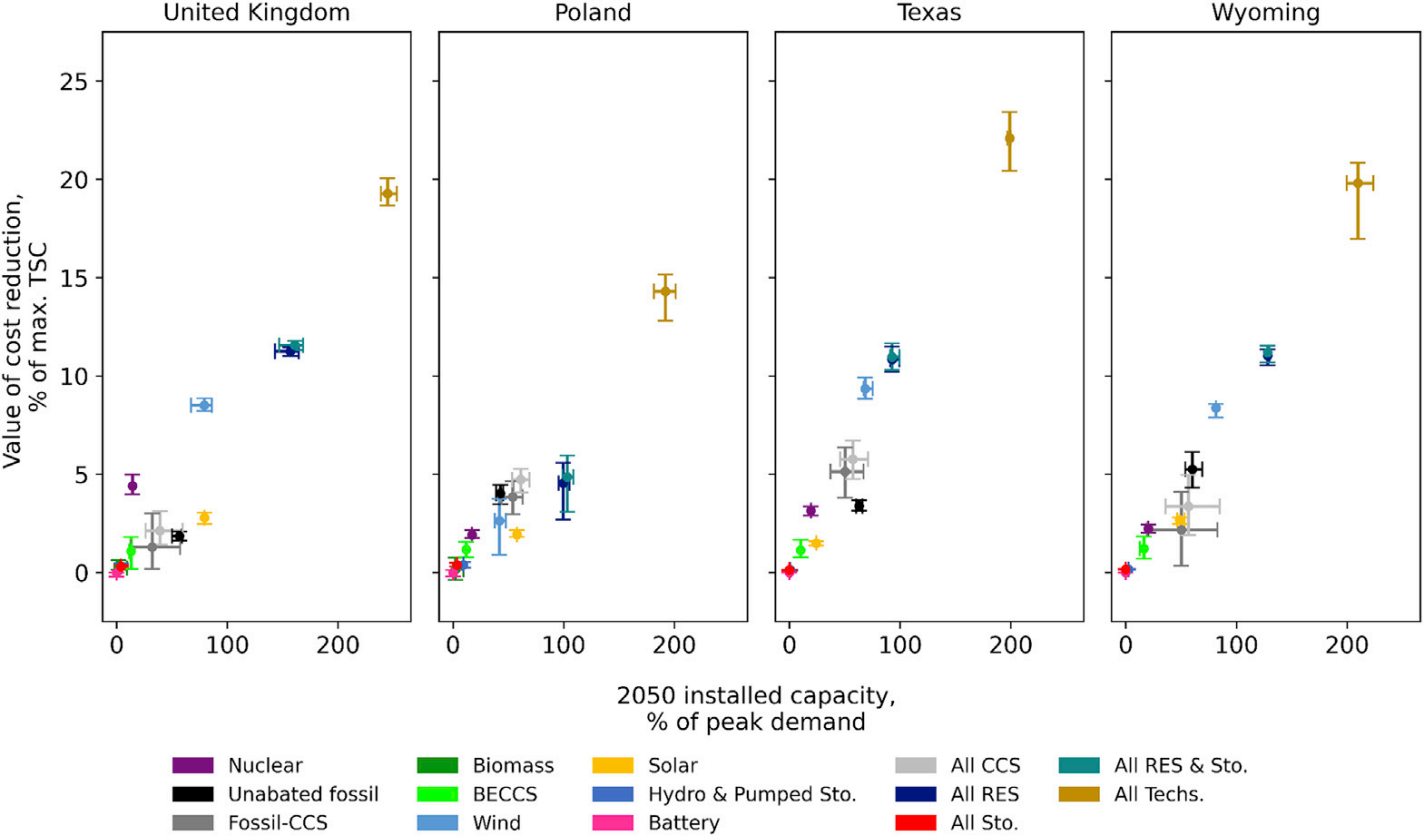
Country-specific impact of technology capital cost reduction on total system cost The color of the plot indicates the group of technologies’ capital costs being reduced while the others are held constant. Ranges of optimal capacities in 2050 and values of cost reduction are provided in each corresponding plot/group.

In line with the net-zero target, the deployment of renewable power is expected to greatly increase, particularly for onshore wind, owing to a more reliable supply compared to solar PV and a cheaper capital cost compared to offshore wind. As previously discussed, the increased capacity is inflated to compensate for its intermittency. Accordingly, further cost reduction of onshore wind can significantly reduce the total system cost. This, however, does not apply to Poland, owing to the moratorium on onshore wind deployment policy in the country. As shown in Figure 5, cost reduction of wind (27%) and solar PV (47%) may lower the range of TSCs by 10%–13% in the UK, Texas, and Wyoming, with wind cost reduction alone leading to an 8%–10% TSC reductions in those systems. As can be seen, solar PV contributes only marginally to the reduction in TSC, owning to a low capacity share and an already low capital cost, respectively. Surprisingly, the impact of cost reduction on electricity storage does not follow the trend observed in wind turbines. This is because the availability of flexible low-carbon power plants, such as CCS, appreciably reduces the role of storage.

In contrast to wind and solar PV, the impact of fossil plants’ cost reduction on system costs is unique to each system. In the UK, the combination of high fuel price, highly seasonal load profile, and significant expansion of wind and solar PV place flexibility service on a high priority. To provide this service, the cost of fossil-based power plants at £ 450–3200/kW is already low compared to other alternatives, such as nuclear and battery. In addition, the installed capacity that is not more than 20% of peak load decreases the value of technology cost reduction. For instance, as shown in Figures 5, 32%–45% CCS cost reduction can only reduce the range of system costs by 1%–3%. In contrast, the value of nuclear cost reduction is higher, between 4%–5% of BAU’s system costs, as the cost of the technology in the UK is among the most expensive worldwide.

Compared to the UK, a significant capacity of thermal plants, including a considerable share of CCGT-CCS, can be maintained in Texas, owing to cheap natural gas. Hence, the value of CCS cost reduction is 2–3 times the value in the UK, *i*.*e*., 4%–6% of BAU. By contrast, Wyoming’s demand profile tends to be flat as the demand is dominated by the industrial sector. As a result, the deployment of low carbon technologies which operate akin to baseload manner, such as nuclear and CCS, is significant. Capacities of nuclear and CCS in Wyoming in 2050 are ∼34% and 46%–86% of the peak demand, which are higher than that in the UK (8% and 24%–56%) and Texas (14% and 52%–74%). However, since CCS and nuclear are already relatively cheap in the US (IEA/OECD, 2018), the value of cost reduction of the technologies is downgraded.

Despite having a similar demand profile to Wyoming, natural gas in Poland is expensive while further onshore wind deployment is banned. Owing to offshore wind costs and PV intermittency, the country is expected to rely heavily on thermal plants, particularly CCS. Hence, with the 2050 installed capacity at 64%–72% of peak demand, CCS cost reduction can lower system costs by 3%–4%.

These results also reveal that technologies with low marginal costs, such as nuclear, wind, and solar power, benefit from capital cost reduction. However, in contrast to wind or solar power, the expansion of nuclear capacity is primarily constrained by its historically low build rate. Hence, technology improvements aiming at lowering the capital cost are expected to be particularly valuable for wind, rather than for nuclear. Similarly, the system value of CCS emerges as being sufficiently high relative to competing options such that further reductions in CCS capital cost do not have a material impact on overall system costs.

**Figure 6:**
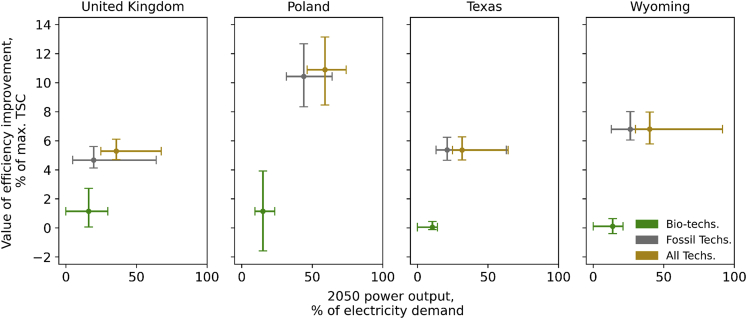
Impacts of thermal plants efficiency gains on total system cost Each plot shows the ranges of optimal power output in 2050 (%) and the value of efficiency gains (%) for each group of thermal power plants.

Similar to capital cost reductions, Figure 6 shows that the value of thermal plants’ efficiency improvements is system-specific, depending on the future diversification of the technology portfolio and access to cheap fuel prices. As previously discussed, providing flexible and dispatchable capacity is a key role for fossil plants in the UK. Consequently, efficiency improvement is less appreciated due to less fuel consumption. Similarly, owing to cheap natural gas, the value of efficiency improvement in Texas and Wyoming falls within the same range as the UK’s at 3%–5% of the system costs.

The decarbonization of the power system in Poland is challenged by numerous factors. In fact, the prohibition of new onshore wind deployment, PV intermittency, and expensive offshore wind requires the country to heavily rely on coal- and gas-CCS. Despite being abated, coal-CCS carbon intensity at 85–124 kgCO_2_/MWh is still relatively high. Conversely, the price of natural gas in Poland is expensive. Hence, thermal plant efficiency improvement for fossil technologies is key in Poland. Our results show that by improving fossil plants’ efficiency, 8%–14% of TSC reductions can be achieved.

### Conclusion

The 1970s oil shocks and efforts to mitigate climate change have fostered the deployment of various types of new and clean technologies in the electricity system. While this allows the system to be robust against potential shocks, it also means the system value of individual technology innovation diminishes and varies significantly between regions. In contrast, technology innovations which are coherent with the system’s net-zero transitions are more appreciable. Hence, policies need to be tailored accordingly to meet the net-zero target effectively.

Given that a strict emission limit seems not to be the path being chosen by the UK, EU, and the US, we have shown that while a carbon tax is necessary to sustain the deployment of wind, solar PV, and low carbon thermal plants, its value is context-specific. In Poland, a low carbon tax is sufficient to force the system to decarbonize owning to the higher capacity factor of fossil plants and the presence of inefficient coal plants. In contrast, systems with high renewable penetration and high flexible capacity requirements, such as the UK and Texas, require higher carbon tax to allow CCS to be competitive against unabated technologies at a lower capacity factor. Nevertheless, the combination of the carbon tax (stick) and negative emissions credit (carrot) proves to be necessary and sufficient to simultaneously minimize residual emissions and secure an adequate level of CO_2_ removal service, which are keys to delivering net-zero emissions target with high certainty (*i*.*e*., probability greater than 90%).

In line with policies and technology roles needed in each system, advancing technology cost reduction for renewable power, particularly for onshore wind, should be a global priority owing to its future capacity that is inflated by overcapacity requirements. In contrast, dispatchable, low-carbon/zero-carbon technologies such as power-CCS are already at a cost point where the value proposition is robust in systems with high renewable penetration, and as such further cost reduction and efficiency improvement are not a priority. It is hence clear that policy and technology innovation priorities need to be coordinated to align with the system’s characteristics in net-zero transitions.

### Limitations of the study

This study provides an analysis of the effectiveness of policy and technological approaches, namely carbon tax, negative emissions credit, and techno-economic improvements of power generation and storage technologies, in cost-effectively delivering net-zero emissions targets.

In conducting the analysis, we assumed that the electricity system is a static system, in which the parameters assumed in the study, such as techno-economic assumptions, system demand, and fuel availability, do not evolve as decision variables varies in different scenarios. While our previous study (Heuberger et al., 2017) show that the qualitative results remain consistent, it may affect the quantitative outcomes, such as installed capacity and the power output. Although this is not the focus of this study, further study may need to incorporate this aspect, particularly in cases where interaction between the electricity sector and other sectors/systems is emphasized. For instance, the use of BECCS in the electricity system has a potential to provide CDR service to other sectors. However, the overuse of BECCS may reduce the availability of sustainable biomass resources which, in turn, reduce the net CDR service BECCS can provide for each MWh of electricity it generates (Patrizio et al., 2021).

In addition to that, this study employed the ESO framework that, similar to a large number of other optimization models, assume a monopolistic social planner. Despite its capability to provide valuable insights on optimal long-term capacity and unit commitment planning, the system is optimized according to the social planner’s point of view. As a result, such an approach cannot capture different and often conflicting objectives of different actors in the system, particularly in regions or countries which implement a competitive electricity market. Thus, interaction of actors within the system and interconnection between the electricity sector with other sectors would be focuses of the work going forward.

## STAR★Methods

### Key resources table

**Table:** 

REAGENT or RESOURCE	SOURCE	IDENTIFIER
**Deposited data**		

Hourly electricity demand (UK & Poland)	ENTSO-E	https://www.entsoe.eu/data/power-stats/
Hourly electricity demand (Texas & Wyoming)	The U.S. Energy Information Administration (EIA)	https://tinyurl.com/d44jm8wx
Renewable Availability	Renewables Ninja	https://www.renewables.ninja/
Existing capacity (UK)	UK Department for Business, Energy & Industrial Strategy (BEIS)	https://tinyurl.com/ahuv6w9a
Existing capacity (Poland)	The Ministry of State Assets of Poland	https://tinyurl.com/ye2by94m
Existing capacity (Texas & Wyoming)	The U.S. Energy Information Administration (EIA)	https://www.eia.gov/electricity/data/eia860/
Technology costs	UK Department for Business, Energy & Industrial Strategy (BEIS)	https://tinyurl.com/49nsxx5j

**Software and algorithms**		

Electricity Systems Optimization (ESO) framework	Imperial College London	https://zenodo.org/record/1048943#.Yy3IbXZBxPY (https://doi.org/10.5281/zenodo.1048943) https://zenodo.org/record/1212298#.Yy3I5nZBxPY (https://doi.org/10.5281/zenodo.1212298)

### Resource availability

#### Lead contact

Further information and requests for resources should be directed to and will be fulfilled by the lead contact, Niall Mac Dowell (niall@imperial.ac.uk).

#### Materials availability

This study did not generate new materials.

### Method details

In this study, we employed Electricity Systems Optimization (ESO) framework to optimize the total system cost of a given electricity system transitions between 2020 and 2050, as can be illustrated in Figure S1 in the supplemental information, where the original formulation of ESO was described by Heuberger et al. (Heuberger et al. (2017; 2017; 2018). ESO is a capacity expansion planning integrated with a unit commitment model to capture hourly operations of power generation and storage technologies in the system to meet all systems requirements, subject to the technical limitations of each technology in the system. In ESO, system-wide characteristics, such as existing capacity, the system reserve, and inertia requirements, along with hourly parameters, such as the electricity demand, renewable availability, and imported electricity price, are taken into account. Here, the existing capacity and hourly electricity demand profile in 2020 can be seen in Figures S2 and S3 in the supplemental information. To minimize computational expenses while preserving the model validity, the hourly input data is clustered into 11 representative days using the k-means clustering technique. We chose this approach based on our previous study using the same model which compared computational time and results’ error across different numbers of days represented in the model (Heuberger et al., 2017).

Here, power generation and storage technologies are characterized by a set of techno-economic parameters of the technology, namely capital cost, fixed- and variable- operating costs, emissions factor, unit capacity, *etc*. To capture future uncertainties, system optimization in this study is performed under a range of fuel price, technology cost, and thermal efficiency assumptions, where the details of these assumptions are shown in the supplemental information. Here, techno-economic parameters of power generation and storage technologies were treated as exogenous variables, *i*.*e*., the cost and performance of technology are not affected by its deployment. Here, we assume that the cost of technologies to be constant from the first period it is made available for the system. Rather than evaluating the impact of technology cost evolution over periods, the systems transitions are evaluated across different combinations of technology cost levels, that are low, central, and high estimates. Accordingly, our results present the upper bound of the technology learning curve effects. Similarly, fuel price is also not influenced by its demand from system optimization results. We assume that fuels are always available to purchase at the assumed prices and the dynamics of their availability are out of the scope of this study. Moreover, the hourly demand is assumed to be inelastic to the cost of providing the electricity, and the role of demand-side response is not taken into account. Finally, optimization is performed under a monopolistic paradigm, which means that competing interests among different actors in the system are not considered. Techno-economic assumptions we used in this study are presented in Tables S1 and S2 while fuel prices assumptions are available in Tables S3–S5 in the supplemental information.

In this study, we considered a range of power generation and storage technologies for the system. These technologies include nuclear, abated and unabated coal, gas, and biomass technologies, RES technologies (Wind-Onshore, -Offshore, and Solar PV), daily electricity storage (Battery and Pumped Hydro storages), as well as interconnection with the neighboring systems. Here, we limit our scope within the context of power system decarbonization. Therefore, we only include BECCS as an archetype of carbon dioxide removal (CDR) technologies. While other CDR technologies, such as DACS, might add value to the whole economy to remove emissions from other sectors(Daggash et al., 2019; Blanford, 2021; Haszeldine et al., 2018; Fuhrman et al., 2021; Kato and Kurosawa, 2021), we omit these technologies from the model to keep the model tractable for the Monte Carlo analysis. In addition, other studies also suggest that, within the context of power sector decarbonization, DACS and other CDR technologies need to be significantly cheaper to compete against BECCS which is cheaper and can provide a wider range of services for the sector (Daggash et al., 2019). Similarly, as we allow dispatchable thermal plants to be deployed, *i*.*e*., not exclusively optimizing the system toward a high RES target, the role of inter-seasonal storage technologies might be limited. In this context, long-duration electricity storage technologies need to be extremely cheap, *i*.*e*., 10–50 times cheaper than current costs, to add value to the system (Sepulveda et al., 2021; Jenkins and Sepulveda, 2021). Accordingly, here we only consider daily electricity storage to be deployed.

To capture the impact of different systems characteristics, we used the UK, Poland, Texas, and Wyoming as case studies. For each case, a range of carbon tax and negative emissions credit combinations is imposed to quantify its effectiveness and impact on the system transitions, covering 90,720 scenarios that are evaluated in this study. Here, we allow combinations of different carbon tax and negative emissions credit values to reflect a variety of options different countries can choose to decarbonize their energy systems. For instance, the UK and EU strongly support carbon tax mechanisms while the US tends to oppose the approach and, instead, prefers to directly incentivize removal(Daggash and Mac Dowell, 2019; Gugler et al., 2021; Edwards and Celia, 2018; Global CCS Institute, 2019). The value of carbon tax and negative emissions credit every period for each trajectory can be seen in Table S6 in the supplemental information.

## Data Availability

• The attached supplemental information includes all dataset generated or analyzed to perform this study • This study does not report original code • Any additional information is available from the lead contact upon request

## References

[bib1] Bednar J., Obersteiner M., Wagner F. (2019). On the financial viability of negative emissions. Nat. Commun..

[bib2] BEIS (2016).

[bib3] BEIS (2018).

[bib4] Blanford G.J. (2021). Impact of carbon dioxide removal technologies on deep decarbonization of the electric power sector. Nat. Commun..

[bib5] Bouzguenda M., Rahman S. (1993). Value analysis of intermittent generation sources from the system operations perspective. IEEE Trans. Energy Conversion.

[bib6] Daggash H.A., Mac Dowell N. (2019). Higher carbon prices on emissions alone will not deliver the paris agreement. Joule.

[bib7] Daggash H.A., Heuberger C.F., Mac Dowell N. (2019). The role and value of negative emissions technologies in decarbonising the UK energy system. Int. J. Greenh. Gas Control.

[bib8] Dennig F., Budolfson M.B., Fleurbaey M., Siebert A., Socolow R.H. (2015). Inequality, climate impacts on the future poor, and carbon prices. Proc. Natl. Acad. Sci. USA.

[bib9] ECIU (2021). Net zero emissions race. https://eciu.net/netzerotracker/map.

[bib10] Edwards R.W.J., Celia M.A. (2018). Infrastructure to enable deployment of carbon capture, utilization, and storage in the United States. Proc. Natl. Acad. Sci. USA.

[bib11] European Commission (2020).

[bib12] Frew B., Sergi B., Denholm P., Cole W., Gates N., Levie D., Margolis R. (2021). The curtailment paradox in the transition to high solar power systems. Joule.

[bib13] Fuhrman J., Clarens A.F., McJeon H., Patel P., Ou Y., Doney S.C., Shobe W.M., Pradhan S. (2021). The role of negative emissions in meeting China’s 2060 carbon neutrality goal. Oxford Open Clim. Change.

[bib14] Global CCS Institute (2019).

[bib15] Gugler K., Haxhimusa A., Liebensteiner M. (2021). Effectiveness of climate policies: carbon pricing vs. subsidizing renewables. J. Environ. Econ. Manage..

[bib16] Haszeldine R.S., Flude S., Johnson G., Scott V. (2018). Negative emissions technologies and carbon capture and storage to achieve the Paris Agreement commitments. Philos. Trans. A Math. Phys. Eng. Sci..

[bib17] Heuberger C.F., Staffell I., Shah N., Dowell N.M. (2017). A systems approach to quantifying the value of power generation and energy storage technologies in future electricity networks. Comput. Chem. Eng..

[bib18] Heuberger C.F., Staffell I., Shah N., Mac Dowell N. (2017).

[bib19] Heuberger C.F., Rubin E.S., Staffell I., Shah N., Mac Dowell N. (2017). Power capacity expansion planning considering endogenous technology cost learning. Appl. Energy.

[bib20] Heuberger C.F., Staffell I., Shah N., Mac Dowell N. (2018). Impact of myopic decision-making and disruptive events in power systems planning. Nat. Energy.

[bib21] IEA (2019).

[bib22] IEA/OECD (2018).

[bib23] International Atomic Energy Agency (IAEA) (2019).

[bib24] Jenkins J.D., Sepulveda N.A. (2021). Long-duration energy storage: a blueprint for research and innovation. Joule.

[bib25] Kaneko S. (2016). Integrated coal gasification combined cycle: a reality, not a dream. J. Energy Eng..

[bib26] Kato E., Kurosawa A. (2021). Role of negative emissions technologies (NETs) and innovative technologies in transition of Japan’s energy systems toward net-zero CO_2_ emissions. Sustain. Sci..

[bib27] Lamont A.D. (2008). Assessing the long-term system value of intermittent electric generation technologies. Energy Econ..

[bib28] Lehtveer M., Fridahl M. (2020). Managing variable renewables with biomass in the European electricity system: emission targets and investment preferences. Energy.

[bib29] McDonald A., Schrattenholzer L. (2002). Learning curves and technology assessment. Int. J. Technol. Manag..

[bib30] McNerney J., Doyne Farmer J., Trancik J.E. (2011). Historical costs of coal-fired electricity and implications for the future. Energy Pol..

[bib31] Mitchell C. (2016). Momentum is increasing towards a flexible electricity system based on renewables. Nat. Energy.

[bib32] Patrizio P., Pratama Y.W., Dowell N.M. (2020). Socially equitable energy system transitions. Joule.

[bib33] Patrizio P., Fajardy M., Bui M., Dowell N.M. (2021). CO_2_ mitigation or removal: the optimal uses of biomass in energy system decarbonization. iScience.

[bib34] Patt A., Lilliestam J. (2018). The case against carbon prices. Joule.

[bib35] Pratama Y.W., Mac Dowell N. (2019).

[bib36] Pratama Y.W., Mac Dowell N. (2020).

[bib37] REN21 (2021).

[bib38] Rosenbloom D., Markard J., Geels F.W., Fuenfschilling L. (2020). Why carbon pricing is not sufficient to mitigate climate change—and how “sustainability transition policy” can help. Proc. Natl. Acad. Sci. USA.

[bib39] Sepulveda N.A., Jenkins J.D., Edington A., Mallapragada D.S., Lester R.K. (2021). The design space for long-duration energy storage in decarbonized power systems. Nat. Energy.

[bib40] Smil V. (2000). Energy in the twentieth century: resource, conversions, costs, uses, and consequences. Annu. Rev. Energy Environ..

[bib41] Sovacool B.K. (2013). Energy policymaking in Denmark: implications for global energy security and sustainability. Energy Pol..

[bib42] Stiglitz J.E., Stern N., Duan M., Edenhofer O., Giraud G., Heal G., la Rovere E.L., Morris A., Moyer E., Pangestu M. (2017).

[bib43] The Government of the Republic of Korea (2020).

[bib44] The Ministry of Energy of Poland (2018).

[bib45] The Ministry of State Assets of Poland (2019).

[bib46] UK Parliament (2008).

[bib47] UK Parliament (2019).

[bib48] UNFCCC (1992).

[bib49] UNFCCC (1998).

[bib50] UNFCCC (2015).

[bib51] US., EIA (2021). Electric power monthly. https://tinyurl.com/2w5cpe2d.

[bib52] US. EIA, (2013). Levelized Cost of Electricity and Levelized Avoided Cost of Electricity Methodology Supplement.

[bib53] USCB (1976).

[bib54] White House T. (2021). FACT SHEET: President Biden sets 2030 greenhouse gas pollution reduction target aimed at creating good-paying union jobs and securing U.S. leadership on clean energy technologies. https://tinyurl.com/9rsdyx4w.

[bib55] World Bank (2019).

[bib56] Zappa W., Junginger M., van den Broek M. (2019). Is a 100% renewable European power system feasible by 2050?. Appl. Energy.

