# Supplementary material for: National priorities in the power system transition to net-zero: No one size fits all
Source: iScience. 2022 Oct 2;25(10):105260. doi: 10.1016/j.isci.2022.105260 (PMC9579019; doi:10.1016/j.isci.2022.105260)
Supplement: Document S1. Figures S1–S3 and Tables S1–S6 [file mmc1.pdf]

iScience, Volume *25*

Supplemental information

National priorities in the power system transition to net-zero: No one size fits all

Yoga Wienda Pratama, Piera Patrizio, and Niall Mac Dowell

SUPPLEMENTAL INFORMATION 1: ELECTRICITY SYSTEMS OPTIMIZATION (ESO) FRAMEWORK

Nomenclature

Sets		IF	Regional cost factor
i	Technology	Disc	Discounted factor
ig	Power generation technology	Δa	Periodic time steps (years)
is	Electricity storage technology		
a	Year		
c	Representative/clustered day	***Variables***	
t	Hour	sc	Total system cost (£)
		anOPEX	Annual O&M cost (£/year)
Parameters		p	Power output (MWh)
CAPEX	Capital cost (£/MW)	s2d	Storage output to demand (MWh)
OPEXFix	Fixed O&M cost (£/MW.yr)	p2d	Generator output to demand (MWh)
OPEX	Variable O&M (£/MWh)	slak	Unmet demand (MWh)
OPEXSU	Start-up cost (£/MW)	b	Number of units built
OPEXNL	No-load cost (£/MW.h)	u	Number of units started-up
ImpElecPr	Price of imported electricity (£/MWh)	n	Number of power generator units online
VoLL	Value of Lost Load (£/MWh)	o	Number of storage units online
WF	Weight factor of representative days (days)	d	Number of units installed
WFA	Weight factor of remaining-lifetime-adjusted capex (%)	wd	Number of units decommissioned
BRet	Retrofit compatibility (binary)	de	Number of permanently decommissioned units
Dec	Number of units scheduled for decommissioning (units)	brn	Number of units retrofitted at the decommissioning period
LT	Lifetime (years)	brl	Number of units retrofitted a period after the decommissioning period

The analytical approach implemented to perform the analysis in this study can be illustrated in Figure S1. In this study, we employed Electricity Systems Optimization (ESO) framework, where the original formulation of the model has been described by Heuberger et al [1–3]. Here, to capture future uncertainties, a given electricity system is optimized to meet all systems requirements from 2020 until 2050 under a range of fuel price, technology cost, and thermal efficiency assumptions, in which assumptions for these parameters are shown in Supplemental Information 2. We treated all techno-economic parameters of all technologies as exogenous variables. For each case study, 18,144 scenarios with a uniform probability for each scenario were evaluated, where a range of carbon tax and negative emissions credit combinations is implemented to quantify its impact on the system transitions. To capture the impact of different systems characteristics, we used the UK, Poland, Texas, and Wyoming as case studies.

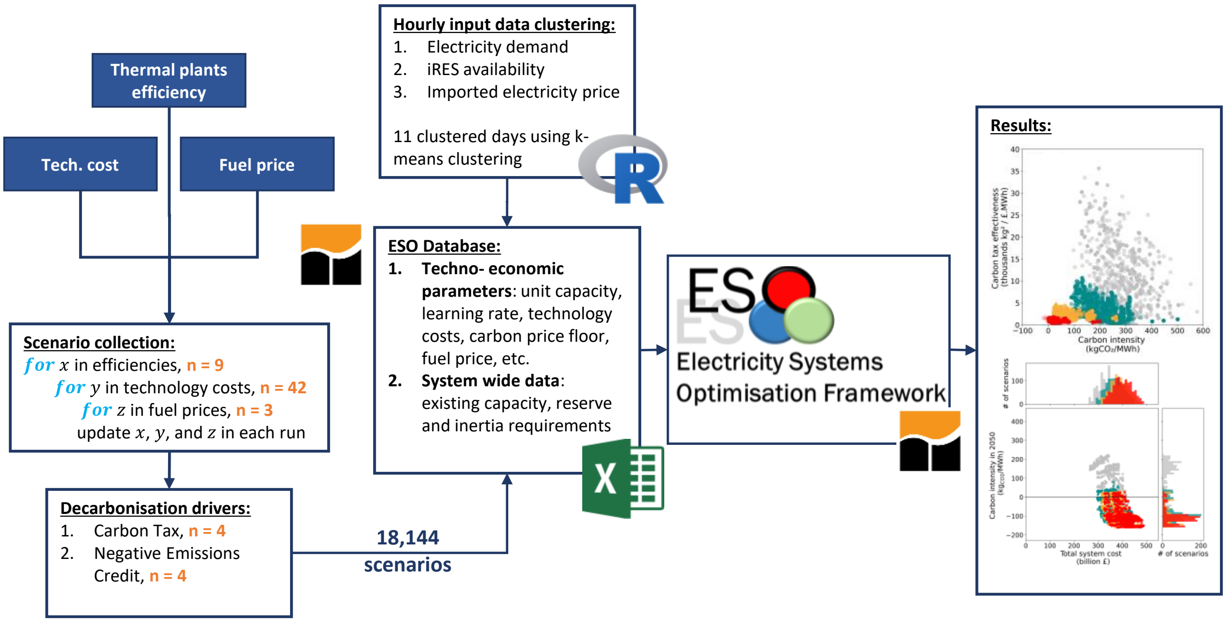

Figure S1. Model Workflow, Related to STAR Methods

In this study, we modify the objective function of the original model by adopting the trapezium rule to capture the annual operating and maintenance costs within the 5-yearly planning period used in the ESO framework. Here, the objective function in the model is described by equations S1 and S2:

$$tsc = \sum_{i,a>1} \left(CAPEX_{i,a}\, WFA_{i,a}\, IF_i\, b_{i,a}/Disc_a\right) + \frac{1}{2}\Delta a \left(anOPEX_{a=1} + anOPEX_{a=7} + 2 \sum_{1<a<7} anOPEX_a \right) \qquad \text{S1}$$

$$\begin{aligned} anOPEX_a = & \sum_{ig,c,t} u_{ig,a,c,t}\, OPEXSU_{ig}\, WF_c/Disc_a \\ & + \sum_{ig,c,t} \left(p_{ig,a,c,t}\, OPEX_{ig,a}\, WF_c + n_{ig,a,c,t}\, OPEXNL_{ig,a}\, WF_c\right)/Disc_a \\ & + \sum_{is,c,t} \left(s2d_{is,a,c,t}\, OPEX_{is,a}\, WF_c + o_{is,a,c,t}\, OPEXNL_{is,a}\, WF_c\right)/Disc_a \qquad \text{S1} \\ & + \sum_{c,t} \left(p2d_{\prime InterImp\prime,a,c,t}\, ImpElecPr_{c,t}\, WF_c + slak_{a,c,t}\, VoLL\, WF(c)\right)/Disc_a \\ & + \sum_{i} d_{i,a}\, OPEXFix_{i,a}/Disc_a \end{aligned}$$

In the original model, all newly installed capacity was assumed to be greenfield assets where existing assets can only be decommissioned once the cost of maintaining the assets overweight their values. In this study, the model has been extended to allow existing fossil and biomass assets to be retrofitted with CCS, thus allowing existing assets, such as Coal, CCGT, and Biomass power plants, to compete in the systems with stricter emissions requirements, reflected by a higher carbon tax and negative emissions credit. Accordingly, equations S2-S5 below are added to the ESO framework. As can be observed in S2, the number of installed units d in the period a is the balance between the number of units in the previous period and number of units newly built b and decommissioned wd. The number of units being decommissioned is equal to the number of units permanently retired de and units being retrofitted now brn, and retrofitted a period later brl, as shown by equation S3. Here, we use binary parameter $BRet$ in S4 to ensure that only the corresponding technology can be retrofitted with CCS. Finally, we assume that CCS retrofit requires 15 years of lead time to capture periods required for planning, feasibility study, permitting, design, and construction, as shown in S5.

$$d_{i,a} = d_{i,a-1} + b_{i,a} - wd_{i,a} \quad \text{S2}$$

$$wd_{i,a} = de_{i,a} + brn_{i,a} + brl_{i,a} \quad \text{S3}$$

$$brn_{i,a} + brl_{i,a-1} = \sum_{i'} BRet_{i,i'} b_{i',a} \quad \text{S4}$$

$$brn_{i,a} + brl_{i,a} \leq d_{i,a} - \sum_{a-3 \leq a' \leq a} b_{i,a'} \quad \text{S5}$$

In this study, the number of units being decommissioned is limited by the number of units installed, as described by equation S6. However, the number of decommissioned units should be greater than the number of existing units scheduled for decommissioning Dec and number of units that pass its lifetime (S7).

$$wd_{i,a} \leq d_{i,a-1} \quad \text{S6}$$

$$\sum_{1 \leq a' \leq a} wd_{i,a'} \geq \sum_{1 \leq a' \leq a} Dec_{i,a'} + b_{i,\left(a' - \frac{LT(i)}{\Delta a}\right)} \quad \text{S7}$$

SUPPLEMENTAL INFORMATION 2: TECHNOLOGY COSTS AND PERFORMANCE

Table S1 shows key technology costs and assumptions used in this study. Here, we based our key assumptions mostly using estimates from the UK's Department of Business Energy and Industrial Strategy (BEIS) to ensure that the estimates are internally consistent and are not subject to proponent bias.

Table S1. Key Technology Costs Assumptions, Related to STAR Methods

	CAPEX (thousand £/MW)			Fixed OPEX (£/MW.yr)	Fixed Variable OPEX (£/MWh)[c]	No-load cost (£/MW.h)	Start-up cost (£/MW)	Regional cost factor [b]	Data sources
	Low	Central	High						
Nuclear	3861	5,268	6980	80,340	3.0	6.2	6,700	0.76	4–7
Coal	1550	1,550	1550	39,780	2.5	7.0	398	1.05	4–8
Coal-CCS(R)	2090	2,879	3213	89,700	5.6	7.8	499	1.02	4–7,9–11
Coal-CCS	2941	4,026	4320	89,700	5.6	7.8	499	1.02	4–7,9–11
Bio	1544	1,879	2238	60,060	2.5	6.2	398	1.04	4–7,12
BECCS(R)	1863	2,776	2893	89,700	10.0	7.8	499	1.04	4–7,13
BECCS	3131	4,249	4705	89,700	10.0	7.8	499	1.04	4–7,13
CCGT	450	565	698	10,140	4.3	3.1	101	1	4–7
CCGT-CCS(R)	1418	1,616	2668	39,780	7.4	3.1	101	1	4–7,9–11
CCGT-CCS	1757	2,055	3158	39,780	7.4	3.1	101	1	4–7,9–11
OCGT	748	846	1200	10,140	6.2	0.8	39	1	4–7
Wind onshore	1044	1,474	1972	29,640	1.6	0.0	0	0.89	4,5,7,12
Wind offshore	2523	2,941	3464	80,340	3.0	0.0	0	0.89	4,5,7,12
Solar PV	506	646	897	10,140	0.0	0.0	0	1.2	4,5,7,12
Hydro	839	1,216	1857	49,920	0.0	0.0	0	1.02	4,5,7,12
Interconnection [a]	1001	1,001	1001	0	0.0	0.0	0	1	14,15
Pumped-Hydro	839	1,216	1857	20,280	6.0	0.0	0	1.02	4,5,7,12
Battery (Li-ion)	2600	3,855	4335	29,640	3.0	0.0	0	1	4,5,7,12

(R) : Retrofit
[a] Import/export connection
[b] Ratio of US over European capital costs
[c] For CCS technology, in addition to fixed variable OPEX, we also assumed an additional cost of £10/t_{CO2} for CO_2 transport and storage[16,17]

Table S2. Key Technology Performance Assumptions, Related to STAR Methods

	Efficiency (%) [a]			CO_2 capture rate (%)	Unit size (MW/unit)	Lifetime (years) [b]	Min. Stable generation (%)	Build Rate (Unit/year)				Data sources
	Low	Central	High					UK	Poland	Texas	Wyoming	
Nuclear	100	100	100	-	600	50	75	0.6	0.6	1.6	0.2	[4–6]
Coal	36	42	48	-	500	40	30	1.2	2.6	3.6	1.4	[4–6]
Coal-CCS(R)	26	32	38	90	500	40	30	1.2	2.6	3.0	1.2	[4–6]
Coal-CCS	26	32	38	90	500	40	30	1.2	2.6	3.0	1.2	[4–6]
Bio	36	42	48	-	500	40	30	0.6	0.6	0.6	0.2	[4–6]
BECCS(R)	26	32	38	90	500	40	30	1.8	0.6	2.0	0.2	[4–6]
BECCS	26	32	38	90	500	40	30	1.8	0.6	2.0	0.2	[4–6]
CCGT	53	57	62	-	750	40	50	1.2	0.4	5.8	0.4	[4–6]
CCGT-CCS(R)	46	50	55	90	750	40	30	1.2	0.4	6.0	0.4	[4–6]
CCGT-CCS	46	50	55	90	750	40	30	1.2	0.4	6.0	0.4	[4–6]
OCGT	39	40	42	-	100	40	10	5.0	2.0	5.0	1.6	[4–6]
Wind onshore	100	100	100	-	20	30	-	80.0	10.0	110.0	16.0	[4,5,12]
Wind offshore	100	100	100	-	50	30	-	30.0	16.0	30.0	0.0	[4,5,12]
Solar PV	100	100	100	-	10	30	-	200.0	80.0	80.0	22.0	[4,5,12]
Hydro	100	100	100	-	300	60	-	2.0	0.4	1.0	1.0	[4,5,12]
Interconnection	100	100	100	-	500	50	-	4.0	0.6	0.6	0.0	[14,15]
Pumped-Hydro	80	80	80	-	300	60	-	2.0	0.6	1.0	0.0	[4,5,12]
Battery (Li-ion)	85	85	85	-	100	15	-	15.0	0.6	15.0	3.0	[4,5,12]

[a] HHV efficiency for thermal power generation, round-trip efficiency for storage. Nuclear and renewable costs is in MWh of electricity already, hence 100%.
[b] Physical lifetime
Hourly capacity factors of wind onshore and offshore are obtained from Renewables Ninja [18]

SUPPLEMENTAL INFORMATION 3: EXISTING CAPACITY AND LOAD PROFILE

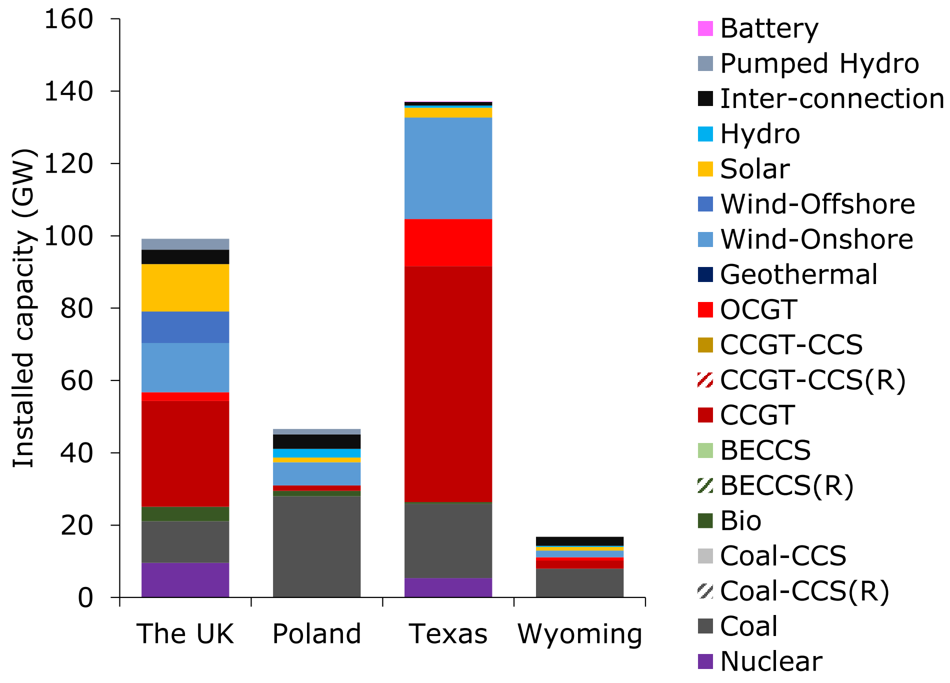

Figure S2. Existing Power Generation Capacity[19–21], Related to STAR Methods

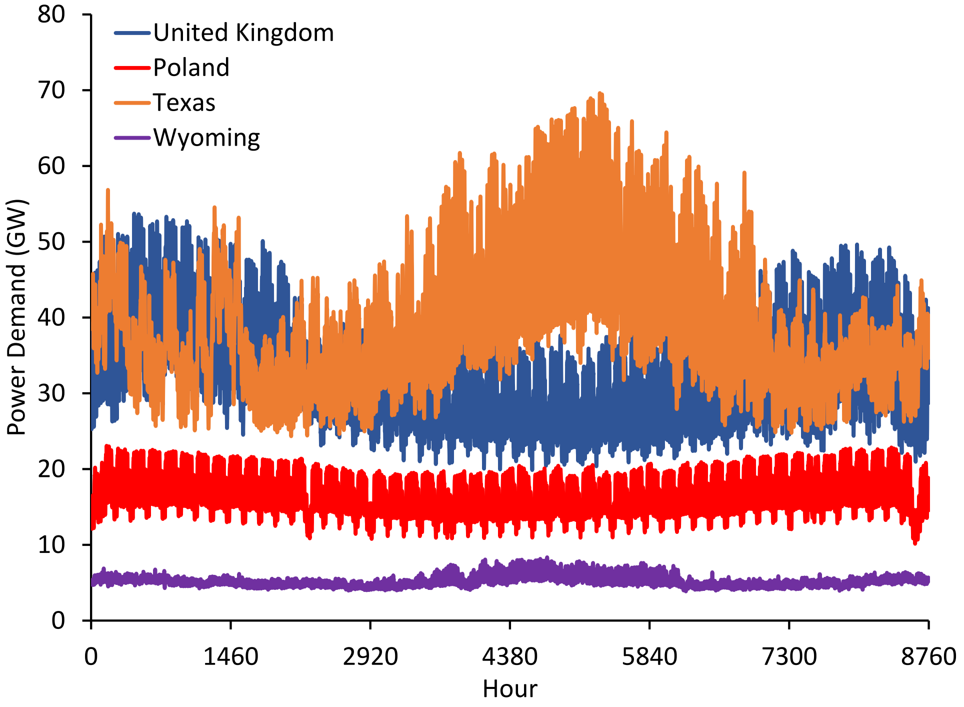

Figure S3. Hourly Electricity Demand[22,23], Related to Figure 1 and STAR Methods

SUPPLEMENTAL INFORMATION 4: FUEL PRICES ASSUMPTIONS

Table S3. Fuel Prices Assumptions for the UK Case[24,25], Related to Figure 1 and STAR Methods

		2020	2025	2030	2035	2040	2045	2050
**Coal**	Low	8.3	6.2	6.8	7.5	7.5	7.5	7.5
	Central	8.3	8.8	9.2	9.9	9.9	9.9	9.9
	High	8.3	11.9	13.1	14.1	14.1	14.1	14.1
**Gas**	Low	16.9	13	14.4	15.5	15.5	15.5	15.5
	Central	16.9	19.1	21.2	23.1	23.1	23.1	23.1
	High	16.9	28.1	29.9	31.7	31.7	31.7	31.7
**Nuclear**	Low	6	4.8	4.8	4.8	4.8	4.8	4.8
	Central	6	6	6	6	6	6	6
	High	6	7.3	7.3	7.3	7.3	7.3	7.3
**Biomass**	-	22.5	22.9	23.3	24.2	25.1	26.0	26.9

Table S4. Fuel Prices Assumptions for Poland Case[25], Related to Figure 1 and STAR Methods

		2020	2025	2030	2035	2040	2045	2050
**Coal**	Low	4.4	4.5	4.7	4.8	5	5.1	5.3
	Central	4.4	4.5	4.7	4.8	5	5.1	5.3
	High	4.4	4.5	4.7	4.8	5	5.1	5.3
**Gas**	Low	12.3	11.8	12	12.1	12.3	12.4	12.6
	Central	12.3	12.4	12.6	12.8	13	13.1	13.3
	High	12.3	13.1	13.3	13.5	13.7	13.8	14
**Nuclear**	Low	6	4.8	4.8	4.8	4.8	4.8	4.8
	Central	6	6	6	6	6	6	6
	High	6	7.3	7.3	7.3	7.3	7.3	7.3
**Biomass**	-	19.3	19.7	20.0	20.8	21.5	22.3	23.0

Table S5. Fuel Prices Assumptions for Wyoming and Texas (the US) Cases[26], Related to Figure 1 and STAR Methods

		2020	2025	2030	2035	2040	2045	2050
**Coal**	Low	5.7	5.2	5.1	5.1	5	4.9	4.8
	Central	5.7	5.5	5.5	5.5	5.5	5.5	5.5
	High	5.7	5.7	5.8	5.8	5.8	5.8	5.9
**Gas**	Low	7.5	7.4	7.8	7.9	7.8	7.7	7.7
	Central	7.5	9.2	10.2	10.2	10.6	10.9	11.3
	High	7.5	10.7	13.9	15.5	16.7	17.8	19.8
**Nuclear**	Low	6	4.8	4.8	4.8	4.8	4.8	4.8
	Central	6	6	6	6	6	6	6
	High	6	7.3	7.3	7.3	7.3	7.3	7.3
**Biomass**	-	15.8	16.1	16.4	17.0	17.6	18.2	18.8

SUPPLEMENTAL INFORMATION 5: CARBON TAX AND NEGATIVE EMISSIONS TRAJECTORIES

To reflect different approaches countries can adopt to drive their electricity system to decarbonize, a scenario based approach was implemented in this study by varying combinations of carbon tax[27] and negative emissions credit[28] trajectories to quantify different role these approaches in the system and how they affect the range of total system costs and 2050 carbon intensities.

Table S6. Carbon Tax and Negative Emissions Credit Assumptions[27,28], Related to Figures 3 and 4 and STAR Methods

		2020	2025	2030	2035	2040	2045	2050
**Carbon tax (£/tCO2)**	Low	*	22	44	67	89	111	133
	Central	*	52	89	126	162	199	236
	High	*	82	132	183	233	283	334
**Negative emissions credit (£/tCO2)**	Low	0	20	22	32	42	52	62
	Central	0	41	44	64	84	104	125
	High	0	81	87	128	168	209	249

* **Note:** Carbon tax in 2020 uses existing carbon tax in each case, that is 18 £/tCO2 in the UK, 12 £/tCO2 in Poland, and 0 t/CO2 in Wyoming and Texas. These numbers are also used throughout the periods for the current carbon tax scenario

SUPPLEMENTAL REFERENCES

1. Heuberger, C.F., Staffell, I., Shah, N., and Mac Dowell, N. (2017). A systems approach to quantifying the value of power generation and energy storage technologies in future electricity networks. Comput. Chem. Eng. *107*, 247–256.
2. Heuberger, C.F., Rubin, E.S., Staffell, I., Shah, N., and Mac Dowell, N. (2017). Power capacity expansion planning considering endogenous technology cost learning. Appl. Energy *204*, 831–845.
3. Heuberger, C.F., Staffell, I., Shah, N., and Mac Dowell, N. (2018). Impact of myopic decision-making and disruptive events in power systems planning. Nat. Energy *3*, 634–640.
4. BEIS (2016). Electricity generation costs.
5. BEIS (2020). Electricity generation costs report 2020 (Department for Business, Energy and Industrial Strategy (BEIS)).
6. Leigh Fisher and Jacobs (2016). Electricity generation costs and hurdle rates.
7. IEA/OECD (2018). World Energy Outlook 2018 (International Energy Agency (IEA)).
8. McNerney, J., Doyne Farmer, J., and Trancik, J.E. (2011). Historical costs of coal-fired electricity and implications for the future. Energy Policy *39*, 3042–3054.
9. Rubin, E.S., Davison, J.E., and Herzog, H.J. (2015). The cost of CO2 capture and storage. Int. J. Greenh. Gas Control *40*, 378–400.
10. Mac Dowell, N., and Staffell, I. (2016). The role of flexible CCS in the UK's future energy system. Int. J. Greenh. Gas Control *48*, 327–344.
11. Ferrari, N., Mancuso, L., and Cotone, P. (2012). Operating flexibility of power plants with CCS.
12. ARUP (2016). Review of renewable electricity generation cost and technical assumptions.
13. Bui, M., Fajardy, M., and Mac Dowell, N. (2017). Bio-Energy with CCS (BECCS) performance evaluation: Efficiency enhancement and emissions reduction. Appl. Energy *195*, 289–302.
14. Junginger, M., Faaij, A., and Turkenburg, W.C. (2004). Cost reduction prospects for offshore wind farms. Wind Eng. *28*, 97–118.
15. DECC (2013). Electricity generation costs 2013.
16. Element Energy, E4tech, and Cambridge Econometrics (2022). Policy mechanisms for first of a kind direct air carbon capture and storage (DACCS) and other engineered greenhouse gas remobals.
17. Summit Power, and Caledonia Clean (2017). Clean Air-Clean Industry-Clean Growth: How Carbon Capture Will Boost the UK Economy.
18. Pfenninger, S., and Staffell, I. (2016). Renewables Ninja. https://www.renewables.ninja/.
19. BEIS (2021). DUKES chapter 5: statistics on electricity from generation through to sales. Dig. UK Energy Stat. Electr. https://tinyurl.com/ahuv6w9a.
20. The Ministry of State Assets of Poland (2019). Executive summary of Poland's national energy and climate plan for the years 2021-2030.
21. EIA (2021). Form EIA-860 detailed data with previous form data (EIA-860A/860B). ELECTRICITY. https://www.eia.gov/electricity/data/eia860/.

22. ENTSO-E (2015). Power statistics. https://www.entsoe.eu/data/power-stats/.
23. EIA Hourly electricity grid monitor. https://www.eia.gov/beta/electricity/gridmonitor/dashboard/electric_overview/US48/US48.
24. BEIS (2019). 2019 Fossil Fuel Price Assumptions (UK Department for Business Energy and Industrial Strategy (BEIS)).
25. Paardekooper, S., Lund, R., Mathiesen, B.V., Chang, M., Petersen, U.R., Grundahl, L., David, A., Dahlbæk, J., Kapetanakis, J., Lund, H., et al. (2015). Heat Roadmap Europe: Quantifying the Impact of Low-carbon Heating and Cooling Roadmaps.
26. EIA (2019). Annual Energy Outlook 2019 (U.S. Energy Information Administration (EIA)).
27. BEIS (2019). Updated short-term traded carbon values used for UK public policy appraisal (Department for Business, Energy & Industrial Strategy (BEIS)).
28. HM Treasury (2018). The green book: Central government guidance on appraisal and evaluation (HM Treasury).
